# Supplementary material for: Integrated rupture mechanics for slow slip events and earthquakes
Source: Nat Commun. 2022 Nov 28;13:7327. doi: 10.1038/s41467-022-34927-w (PMC9705564; doi:10.1038/s41467-022-34927-w)
Supplement: Supplementary file 1 — Supplementory info [file 41467_2022_34927_MOESM1_ESM.pdf]

Integrated rupture mechanics for slow slip events and earthquakes

Huihui Weng[1,2*] and Jean-Paul Ampuero[1]

[1]*Université Côte d'Azur, IRD, CNRS, Observatoire de la Côte d'Azur, Géoazur, 250 rue Albert Einstein, Sophia Antipolis, 06560 Valbonne, France*

[2]*School of Earth Sciences and Engineering, Nanjing University, Nanjing 210023, China*

Correspondence to Huihui Weng (email: weng@nju.edu.cn)

Contents

7 Supplementary Figures

Figure S1.

Figure S2.

Figure S3.

Figure S4.

Figure S5

Figure S6

Figure S7.

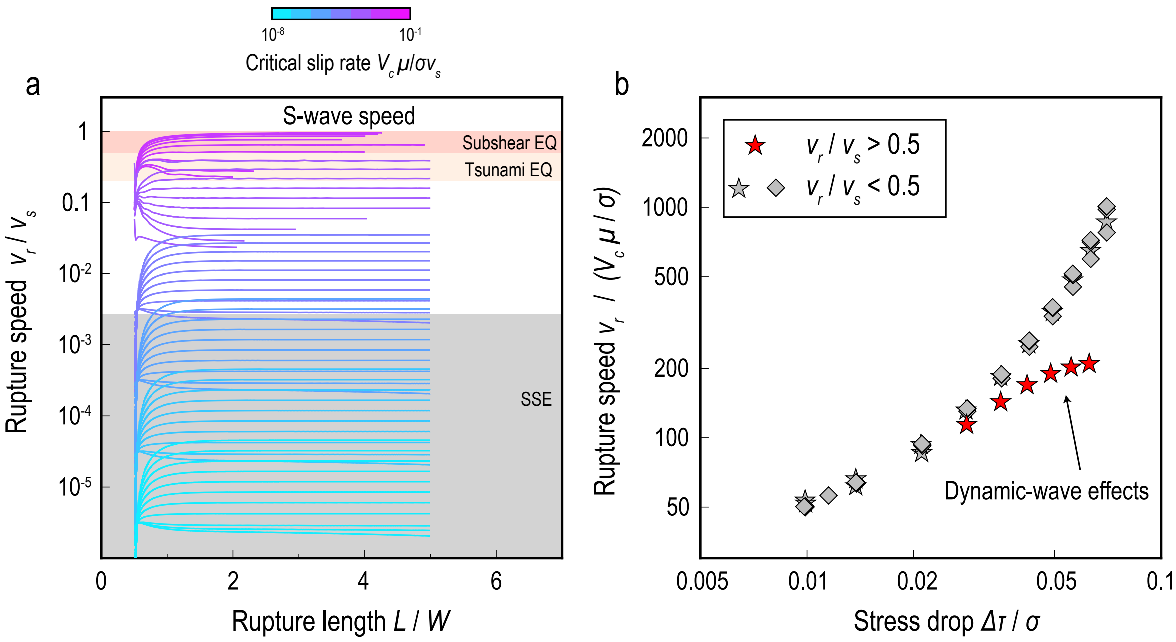

Figure S1: Rupture propagation of SSEs and earthquakes. (a) Coloured curves represent rupture speed as a function of normalized rupture distance based on fully dynamic and quasi-dynamic simulations (coloured curves coded by critical slip rate). (b) Normalized rupture speed (not accounting for the Lorentz contraction factor) versus normalized stress drop for simulated ruptures (legend).

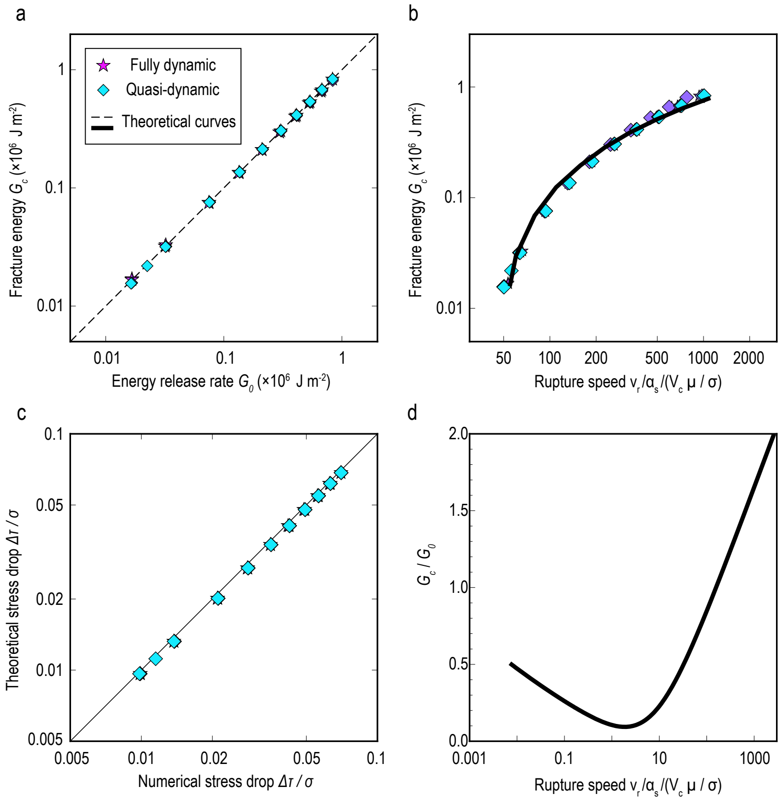

Figure S2: Energies of steady SSE and earthquake ruptures. (a) Symbols represent fracture energy and energy release rate numerically estimated from the fully dynamic and quasi-dynamic simulations (legend). The dashed line indicates the energy balance predicted by theory. (b) Fracture energy versus rupture speed based on simulations and theoretical prediction (black curve). (c) Comparison of stress drop between the numerical and theoretical estimates. (d) An example shows the dependency of G_c/G_0 on rupture speed v_r given an initial shear stress (Methods).

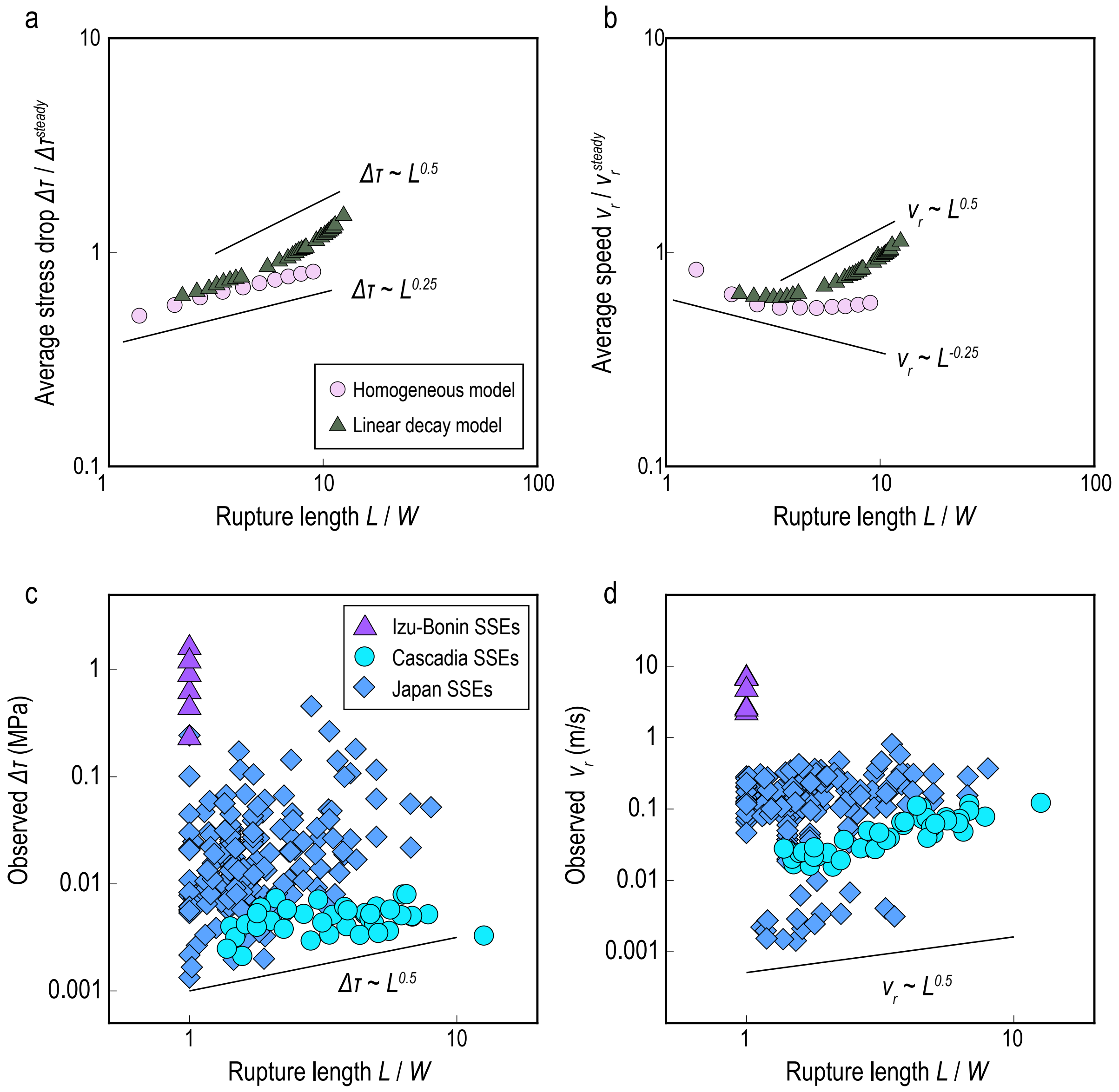

Figure S3: Scaling relations of stress drop and rupture speed. Stress drop (a) and rupture speed (b) as a function of rupture length for the homogeneous model (pink circles) and the linear decay model (green triangles). (c-d) Stress drop and rupture speed of real SSEs from three different regions (indicated in legend) versus normalized rupture length. The stress drop is simply estimated by $\Delta\tau \approx \mu D/W$.

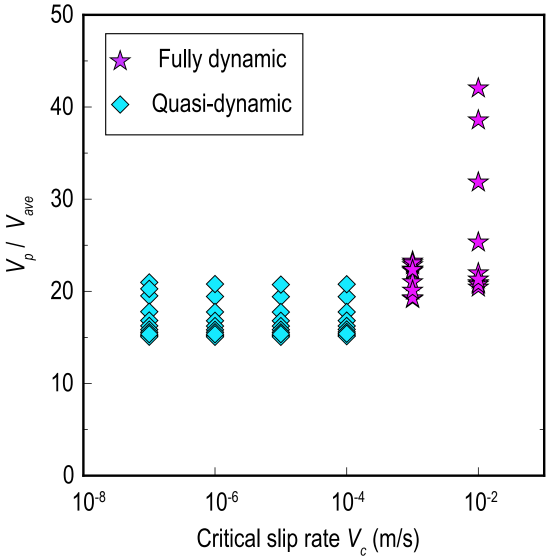

Figure S4: Empirical ratio between peak and average slip rates. Symbols represent the ratio of peak slip rate to average slip rate for various critical slip rates based on fully dynamic (stars) and quasi-dynamic (diamonds) simulations.

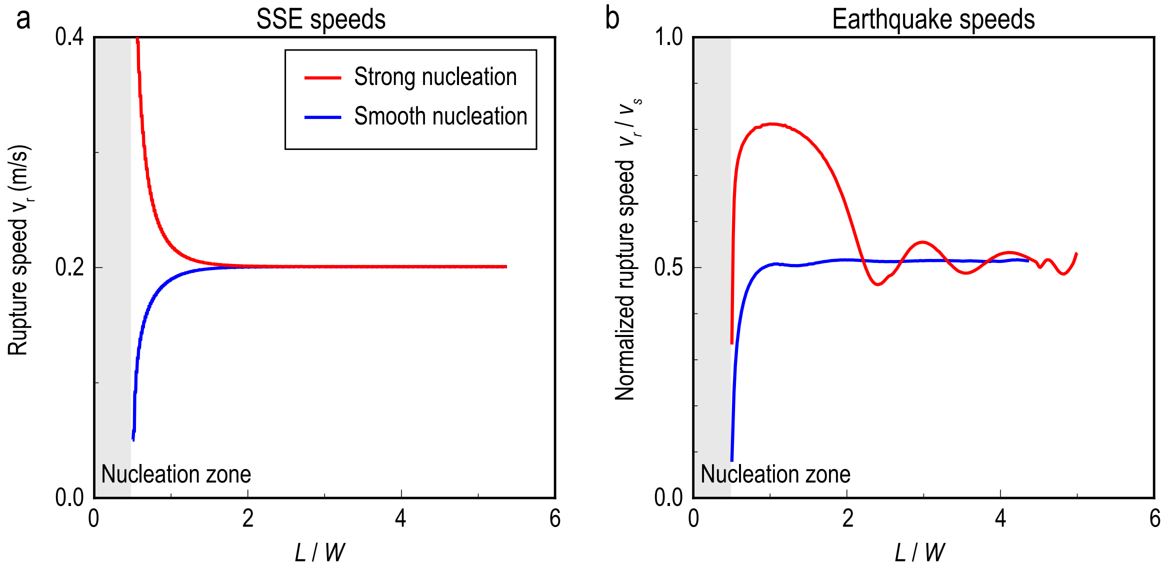

Figure S5: Effects of nucleation conditions on steady rupture propagation. (a) Rupture speeds as a function of normalized distance for two quasi-dynamic SSE simulations with different nucleation strategies: strong overstressed nucleation and smooth nucleation. The grey region marks the nucleation zone. (b) same as (a), but for fully dynamic earthquake simulations.

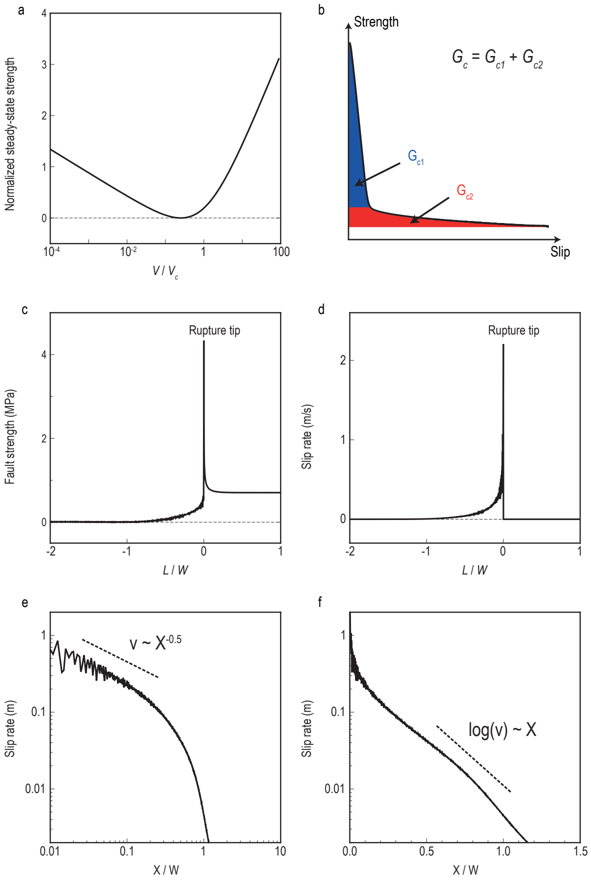

Figure S6: Steady-state fault strength and one example of fault strength and slip rate evolution (a) Normalized steady-state frictional strength as a function of slip rate for V-shape rate-and-state friction with $a/b = 0.8$. (b) The evolution of fault strength as a function of fault slip governed by V-shape rate-and-state friction. The blue region marks the fracture energy caused by the first weakening stage. The red region marks the fracture energy caused by the second weakening stage. (c-d) The snapshots of the fault strength (c) and the slip rate (d) of one dynamic rupture model illustrate the feature of a pulse-like rupture, on a fault with a finite width and controlled by V-shape rate-and-state friction. (e-f) The log-log (e) and semi-log (f) relation between the distance to the rupture tip and slip rate.

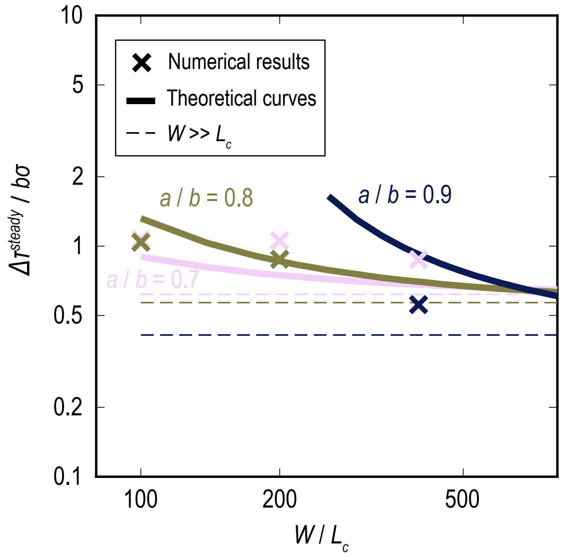

Figure S7: Dependence of critical stress drop for runaway ruptures The critical stress drop $\Delta\tau_{run}/b\sigma$ versus W/L_c for different values of a/b (colours), based on the numerical simulations (cross symbols) and the theoretical predictions (thick and dash curves).
